# Development of muco-adhesive orally disintegrating tablets containing tamarind gum-coated tea powders for oral care

**DOI:** 10.1016/j.ijpx.2019.100012

**Published:** 2019-04-04

**Authors:** Rika Kiniwa, Masaki Miyake, Shin-ichiro Kimura, Shigeru Itai, Hiromu Kondo, Yasunori Iwao

**Affiliations:** a Laboratory of Pharmaceutical Engineering and Drug Delivery Science, School of Pharmaceutical Sciences, University of Shizuoka, 52-1 Yada, Suruga-ku, Shizuoka 422-8526, Japan; b Laboratory of Microbiology and Immunology, School of Pharmaceutical Sciences, University of Shizuoka, 52-1 Yada, Suruga-ku, Shizuoka 422-8526, Japan

**Keywords:** ECG, Epicatechin gallate, EGC, Epigallocatechin, EGCG, Epigallocatechin gallate, GCG, Gallocatechin gallate, ODTs, Orally disintegrating tablets, ODTTs, Orally disintegrating tea tablets, SEM, Scanning electron microscope, Oral care, Orally disintegrating tablet, Tea powder, Microwave, Adhesive property, Tamarind gum

## Abstract

The aim of this study was to design and evaluate muco-adhesive orally disintegrating tablets manufactured by microwave irradiation and containing polysaccharide. We prepared orally disintegrating tea tablets (ODTTs) containing a 1 w/w% mass fraction of one of five polysaccharides (gum arabic, carrageenan, guar gum, tamarind gum, or pectin) and evaluated the swelling degree, tablet hardness, friability, disintegration time, and adhesive properties. All tablets had a swelling degree of about 1 mm, a hardness of over 13 N, and a friability degree of <1%. Tablets containing gum arabic and tamarind gum had disintegration times of 30 s or less and satisfied requirements as orally disintegrating tablets. This could be attributed to their high void contents, which allowed for water penetration. The adhesive properties and particle retention ratios were highest in ODTTs containing tamarind gum, which was thought to be caused by the rapid disintegration and high viscosity of the tamarind gum itself. When we investigated changing the mass fraction of tamarind gum, we found 1 w/w% was most suitable for rapid disintegration and high adhesiveness. The ODTTs containing 1 w/w% tamarind gum showed significant growth inhibition towards *Streptococcus mutans*. Therefore, microwave irradiation technology and addition of tamarind gum could be used to manufacture muco-adhesive orally disintegrating tablets for oral care.

## Graphical abstract

**Figure:**
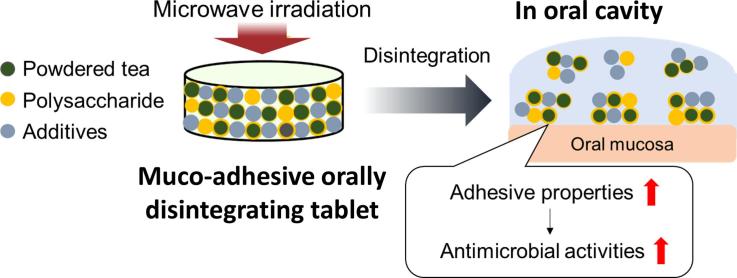

## 1 Introduction

In the modern era, the pneumonia death rate has been increasing with population aging and super-aging societies (Miyashita and Yamauchi, 2018). Over 90% of pneumonia deaths are of older adults aged 65 years or older, and about 70% of older adults suffering from pneumonia have aspiration pneumonia (Teramoto et al., 2008, Baine et al., 2001). Aspiration pneumonia is caused by bacteria or other contaminants that enter the trachea because of decreased swallowing function (i.e., deglutition reflex and cough reflex) (Manabe et al., 2015), which is common in the older adult (Ebihara et al., 2016, Perry et al., 2001, Li et al., 2015). Therefore, it is important to keep the oral cavities of older adults clean to prevent aspiration pneumonia. In addition, effective oral care can prevent intraoral diseases (e.g., periodontal disease and dental caries) and systemic diseases such as angina, brain infarction, and diabetes that can be induced by intraoral disease. Oral care preparations can improve quality of life for older adults (Langmore et al., 1998, Teramoto et al., 2015), especially if the preparations are easily handled and can be taken even by bedridden patients.

Methods used for oral care include tooth brushing, mouthwashes, and oral care supplements to control plaque derived from bacteria. In dental caries, some *Streptococcus mutans* (*S. mutans)* produce insoluble glucan, which various microorganisms adhere to and then generate acid and dissolve the tooth enamel (Leme et al., 2006, Forssten et al., 2010). To prevent dental caries, chlorhexidine has been used to decrease plaque accumulation (Charles et al., 2004). However, some side effects, such as tooth coloration, tartar formation in gingival crevices, and taste disorders, have been reported when it is used for more than 2 weeks. Consequently, it is best avoid long-term use of chlorhexidine where possible (James et al., 2017). As other candidates, polyphenols such as tannins, catechin, quercetin, and anthocyanin, which are biological components of certain plants and very safe, have been reported to inhibit the formation of glucosyltransferase and biofilms involved in the production of insoluble glucan (Ferrazzano et al., 2009, Percival et al., 2006, Yamanaka-Okada et al., 2008). Polyphenols have not only antibacterial activity but also antioxidant and anti-inflammatory activities (Almajano et al., 2008, Jalil and Ismail, 2008, Cavet et al., 2011). In diseases in the oral cavity such as dental caries and periodontal disease, inflammation occurs as a host immune response towards bacteria. The use of polyphenols with antioxidant and anti-inflammatory activities can reduce inflammation caused by oral bacteria and keep the inside of the mouth clean. Tea leaves contain many compounds with antimicrobial, antioxidant, and anti-inflammatory activities, and are a candidate for preparation of oral care products (Graham, 1992).

**Fig. 1:**
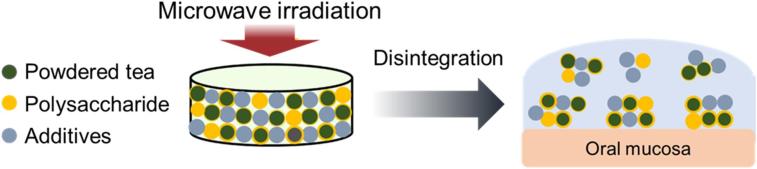
Illustration of the interaction between oral mucosa and powdered tea after tablet disintegration.

We recently developed a novel method for facile preparation of orally disintegrating tablets (ODTs) by microwave irradiation of wet-molded tablets containing mannitol, sugar alcohol, a polymeric disintegrant, and water absorbent materials (Sano et al., 2011, Sano et al., 2013, Sano et al., 2014). ODTs are designed to dissolve in a small amount of water in the oral cavity to enable patients with dysphagia or restricted water intake to swallow tablets. They represent one of the most patient-friendly dosage forms (Wade et al., 2012, Pfister and Ghosh, 2005). In our methodology, the microwave irradiation led to the formation of water vapor, which resulted in the expansion of the pores inside the tablets and the formation of new void networks. These new void networks allowed for the water to penetrate into the tablet more efficiently and resulted in a decrease in their disintegration time. In addition, the formation of water vapor during the microwave irradiation also led to the dissolution/precipitation of some of the mannitol particles, which resulted in the formation of new solid bridges. The formation of new solid bridges between the mannitol particles led to an increase in the hardness of the tablets. Taken together, this new method therefore makes it possible to prepare ODTs with opposing physicochemical properties such as rapid disintegration time and enhanced hardness. In addition, we developed orally disintegrating tea tablets (ODTTs) containing powdered tea leaves. These ODTTs disintegrated within the oral cavity within 30 s (Tanaka et al., 2016). In a preliminary evaluation of antibacterial activity against *S. mutans*, an ODTT extract greatly inhibited bacterial growth compared with artificial saliva. As the disintegrated particles are kept in the oral cavity for a long time, the antimicrobial action is sustained, and this should increase the therapeutic effect for oral care. If we could give ODTTs muco-adhesive properties, the powdered tea that disintegrates in the oral cavity would adhere to the oral mucosa and exhibit sustained antibacterial action against oral microbial flora and provide efficient oral care (Fig. 1).

In the present study, we tried to make muco-adhesive orally disintegrating tea tablets by adding polysaccharides. Polysaccharides contain many connected monosaccharides and have different types of bonding and molecular weights (Wüstenberg, 2015). Polysaccharides contain hydroxyl and carboxyl groups, and their thickening or gelling action is determined by the type and concentration of these groups (Dickinson, 2003). They are used in a wide range of applications in foods, cosmetics, oral care products, and medicines (Avachat et al., 2011, Nayak et al., 2014, Datta and Bandyopadhyay, 2006). Mucin, the main component of oral mucosa, reportedly strengthens interactions with hydrogen bond donors in the solute, and polysaccharides containing many hydroxyl and carboxyl groups interact with mucin (Caron et al., 2015). This allows powdered tea to attach to the oral cavity via polysaccharides. Because we used microwave irradiation to prepare the ODTTs, we selected thermally stable polysaccharides (gum arabic, carrageenan, guar gum, tamarind gum, and pectin). We determined the effects of these polysaccharides on the muco-adhesive properties of the tablets for potential use as oral care preparations.

## 2 Materials and methods

### 2.1 Materials

Oolong tea leaves (Utonrousousuisen) were purchased from the Banboo Cakan Chinese tea ceremony (Kochi, Japan). d(−)-Mannitol was purchased in its β crystalline form from Merck Ltd (Tokyo, Japan). Low-substituted hydroxypropyl cellulose (L-HPC) (mean particle size, 45 µm; hydroxyepoxy group, NBD-020) was supplied by Shin-etsu Chemical Co., Ltd (Tokyo, Japan). Polyvinylpyrrolidone (PVP) (Kollidon® 25) was obtained from BASF Japan (Tokyo, Japan). Gum arabic (Gum arabic HP powder), carrageenan (Sheepy gum® FA), guar gum (Guar pack® PF-20), tamarind gum (Glyroid® 6C), and pectin (H&F pectin classic AF701) were purchased from DSP Gokyo Food and Chemical (Osaka, Japan). Yeast nitrogen base without amino acids was purchased from Nippon Becton Dickinson Co., Ltd (Tokyo, Japan). Glucose, ammonium sulfate, polypeptone S, and monopotassium phosphate were purchased from Wako Pure Chemical Industries, Ltd (Osaka, Japan). Brain heart infusion was purchased from Nissui Pharmaceutical Co., Ltd. (Tokyo, Japan). All other reagents used were of the highest grade available from commercial sources.

### 2.2 Design of muco-adhesive tablet formulations containing polysaccharide-coated tea powders

#### 2.2.1 Formulation of ODTTs containing polysaccharides

ODTTs were prepared using an established method with a slight modification (Tanaka et al., 2016). Oolong tea leaves were ground using a standard tea grinder (Teafine Mutow, Shizuoka, Japan). The resulting powder was sieved through a 75-µm screen and 32 w/w% of the powdered tea was placed in a mortar. A 1.68 M Na_2_HPO_4_ aqueous solution (40 w/w% mass fraction of the powdered tea mass) for use as an adsorption solvent was then dropped into the mortar using a pipette, and the resulting mixture was stirred using a pestle to obtain a homogenous mixture. Polysaccharide (1 w/w% mass fraction of the total mass of mixed powder) was added and it adsorbed onto and coated the powdered tea surface. The ODTT formulations are summarized in Table 1. d-Mannitol (55 w/w% or 56 w/w%), L-HPC (10 w/w%), and PVP (2 w/w%) were then added, and the resulting mixture was blended with the pestle. An additional portion of distilled water (45 w/w% mass fraction of the total mass of mixed powder) was then added to the mortar as a granulation solvent, and the resulting mixture was granulated for approximately 1 min. A portion (60 mg) of the wet granules was compressed using a compression test apparatus (MPC-100, Okada Seiko, Tokyo, Japan) fitted with several punches (ø 5 mm). Considering the further development of tablets which are easily handled and can be taken even by bedridden patients, smaller sized tablet (ø 5 mm) was selected. A compression force of 0.43 kN was used for tablet preparation. The wet molded tablets were heated in a microwave oven (NE-EH226, Panasonic, Osaka, Japan) at 500 W, which gave a reaction temperature of 140 °C. After microwave treatment, the tablets were dried in a thermostatic chamber at 80 °C for 24 h.

**Table 1:** ODTT formulations containing polysaccharides.

		%(w/w)					
		F1	F2	F3	F4	F5	F6
Powdered tea	Utonrousousuisen	32	32	32	32	32	32
Polysaccharide	Gum arabic		1				
	Carrageenan			1			
	Guar gum				1		
	Tamarind gum					1	
	Pectin						1
Excipient	d (−)-Mannitol	56	55	55	55	55	55
Disintegrant	Low-substituted hydroxypropyl cellulose (L-HPC NBD-020)	10	10	10	10	10	10
Binder	Polyyvinylpyrrolidone (PVP Kollidon® 25)	2	2	2	2	2	2
	Total	100	100	100	100	100	100

#### 2.2.2 Formulation of ODTTs with various tamarind gum mass fractions

We formulated ODTTs with various tamarind gum mass fractions (Table 2). The mass fractions of powdered tea, L-HPC, and PVP did not change; therefore, 32 w/w% of the powdered tea, 10 w/w% of L-HPC and 2 w/w% of PVP were used. The preparation method was similar to that described in Section 2.2.1.

**Table 2:** ODTT formulations containing tamarind gum.

		%(w/w)			
		0.5	1	3	5
Powdered tea	Utonrousousuisen	32	32	32	32
Polysaccharide	Tamarind gum	0.5	1	3	5
Excipient	d (−)-Mannitol	55.5	55	53	51
Disintegrant	Low-substituted hydroxypropyl cellulose (L-HPC NBD-020)	10	10	10	10
Binder	Polyyvinylpyrrolidone (PVP Kollidon® 25)	2	2	2	2
	Total	100	100	100	100

### 2.3 Characterization of ODTTs

#### 2.3.1 Swelling degree

The degree of swelling was calculation according to Eq. (1).

(1) $$\text{Swelling degree}\;={\text{Thickness}}_{\text{Treated}}-{\text{Thickness}}_{\text{Untreated}},$$

where Thickness_Treated_ and Thickness_Untreated_ are the thickness of the microwave -treated and untreated tablets, respectively. The thickness of each tablet was measured with a micrometer with a precision of 0.01 mm (CD-20, Mitsutoyo Corporation, Kanagawa, Japan). Three tablets were randomly selected for thickness measurements and the average values were used for subsequent calculations.

#### 2.3.2 Tablet hardness

The tablet fracture strength was defined as the force required to break the tablet by radial compression. The tablet hardness was determined using a tablet hardness tester (PC30, Okada Seiko, Tokyo, Japan). All of these measurements were repeated three times and the average values were calculated.

#### 2.3.3 Friability

The tablet friability was determined using a tablet friability tester (Friabilator, Toyama Sangyo, Osaka, Japan) in accordance with the procedure described in the seventeenth edition of the Japanese Pharmacopoeia (Osei-Yeboah and Sun, 2015). All the measurements were repeated three times and the average values were calculated.

#### 2.3.4 Disintegration time

The disintegration time was measured using an ODT tester (OD-mate, Imotoseisakusyo, Kyoto, Japan). The bottom surface on which the tablets were placed was 4 mm mesh. The tablet was placed in the center of the mesh, and the tester loading tool was placed on the top of the tablet. An aliquot (10 mL) of artificial saliva, which contained distilled water (1 L), NaCl (1.44 g), KCl (1.47 g), and Tween 80 (3 g) and was at 37 °C, was placed in a beaker and stirred at 1000 rpm. The tablet sandwiched between the loading tool and the bottom surface was lowered in the beaker to the predetermined position. The disintegration time was recorded as the period between when the tester arm was lowered to the predetermined position and when the loading tool touched the bottom of the mesh. The start and end points of each measurement were recorded by a photo microsensor. The maximum disintegration time for these experiments was set to 120 s. All of the measurements were repeated three times and the average values were calculated.

#### 2.3.5 Adhesive property of the tablets

The adhesive properties of the tablets were measured using a table-type tensile compression testing machine (Force Tester MCT-2150, A&D Co. Ltd., Tokyo, Japan) (Ikeuchi-Takahashi et al., 2013). The tablet to be analyzed was fixed with double-sided tape to the movable adapter. Then, a mucin layer (10 w/v%) was prepared to the fixed adapter. Artificial saliva (50 μL) was placed on the mucin layer, and the movable adapter with tablet was placed in contact with the mucin layer. After confirming that the tablet was in contact with the artificial saliva and mucin layer and maintaining for 20 s, the movable adapter was raised (Supplemental Fig. 1A). The load at that time was measured and the adhesiveness was calculated from the load and the tablet area using Eq. (2). All of these measurements were repeated three times and the average values were calculated.

(2) $$Adhesiveness=\frac{\mathrm{Load}\;(N)}{\mathrm{Tablet}\;area(m^{2})}$$

#### 2.3.6 Particle retention ratio

Each tablet was pulverized with a mortar and pestle, and 100 mg of the pulverized powders which have same particle size and particle size distribution analyzed by a laser diffraction particle size analyzer (Mastersizer2000; Marvern, Worcestershire, UK)(data not shown), was suspended in 1 mL of artificial saliva. For analysis, a mucin layer (10 w/v%) was prepared on a glass slide. The glass slide was inclined at 15° and the suspension was dropped on it at a rate of 1 mL/min (Supplemental Fig. 1B). The weights of the suspension before and after the test were used to calculate particle retention ratio on the mucin layer (Eq. (3)). All of these measurements were repeated three times and the average values were calculated.

(3) $$Particle\;retention\;ratio=\frac{\mathrm{Before}\;drop\;treatment\;\left(g\right)-After\;drop\;treatment\;\left(g\right)}{\mathrm{Before}\;drop\;treatment\;(g)}$$

#### 2.3.7 Tablet surface

The tablet surface was observed using a scanning electron microscope (Miniscope®TM3030, Hitachi High-Technologies Co., Tokyo, Japan).

#### 2.3.8 Polysaccharide viscosity

The viscosity was measured using a viscometer (RV DV 2 T, EKOKO SEIKI Co., Ltd., Tokyo, Japan) after dissolving 10 mg of each polysaccharide (gum arabic, carrageenan, guar gum, tamarind gum, or pectin) in 1 mL of 1.68 M aqueous Na_2_HPO_4_ with stirring for 1 min at 12 rpm. Measurements were carried out at 25 °C and 80 °C. All of these measurements were repeated three times and the average values were calculated.

#### 2.3.9 Dissolution of epigallocatechin gallate

A dissolution test was performed for polysaccharide-free tablets without microwave (F1-without microwave), ODTTs with microwave (F1), tablets containing 1 w/w% tamarind gum without microwave (F5-without microwave), and ODTTs containing 1 w/w% (mass fraction) tamarind gum with microwave (F5) using the paddle apparatus. Each test was performed by placing 10 tablets in each vessel, which was filled with 150 mL of pH 6.8 phosphate buffer solution and kept at 37 ± 0.5 °C. The paddle speed was adjusted to 50 rpm. Samples were taken after 5, 10 min, 20 min, 30 min, 1 h, and 2 h. The samples were filtered through a filter with a pore size of 0.20 µm and then applied for high-performance liquid chromatography (HPLC) (LC-2010CHT, Shimadzu, Kyoto, Japan) to determine the amounts of epigallocatechin gallate (EGCG). A mixture of methanol (solvent A) and purified water (solvent B), which was deaerated ultrasonically for more than 10 min in advance, was used as the mobile phase with a flow rate of 1.0 mL/min. Quantification was carried out on TSK ODS-80T_M_ (150 mm × 4.6 mm, 5 µm). The column temperature was 40 °C and the sample volume was 5 µL. The detector was set at 280 nm. For the elution program, the proportion of solvent A was increased from 10% to 50% from 0 to 30 min.

In addition, to completely elute the EGCG from the oolong tea leaves, further incubation (100 rpm, 1 h) was performed after dissolution test and the total amount of EGCG was determined. The ratio of EGCG released into the medium (%) was quantitatively obtained by dividing by the total amount of EGCG.

### 2.4 Antibacterial effect

#### 2.4.1 Preparation of test solutions

Five polysaccharide-free tablets without microwave (F1-without microwave), Five ODTTs with microwave (F1), Five tablets containing 1 w/w% tamarind gum without microwave (F5-without microwave), and Five ODTTs containing 1 w/w% tamarind gum with microwave (F5) were incubated in separate 10-mL aliquots of artificial saliva for 1 h at 37 °C. After 10 min of sonication, centrifugation was performed for 20 min. Each supernatant was filtered through a 0.20-μm sterilizing filter to prepare a test solution.

#### 2.4.2 Quantitative analysis of catechins

Quantification of catechins (epicatechin gallate (ECG), gallocatechin gallate (GCG), epigallocatechin (EGC), and EGCG) in the prepared tablets was carried out by HPLC (LC-2010CHT) in the same manner described in Section 2.3.9.

#### 2.4.3 Evaluation of the antibacterial effect

Yeast nitrogen base without amino acids (4 g), glucose (2.5 g), ammonium sulfate (2.5 g) polypeptone S (1 g), monopotassium phosphate (0.5 g), and water (1000 mL) were added to a beaker, and the pH of the resulting mixture was adjusted to 7.1 with a sodium hydroxide solution. This mixture was used as the catechin medium. The catechin medium (8 mL), test solution (1 mL), and *S. mutans* (270 µL) were cultured for 24 h in brain heart infusion in a boiling tube. Thereafter, cultivation was carried out at 37 °C. The turbidity at 651.5 nm was measured at 0, 3, 5, and 7 h using a spectrophotometer (UV-mini-1240, Shimadzu Co.) and used for determination of growth inhibition. The sample containing artificial saliva was designated as the control for comparison purposes. All of these measurements were repeated three times and the average values were calculated.

### 2.5 Statistical analysis

All date were expressed as the mean ± standard deviation. In tablets properties, the statistical significance of differences between ODTTs with microwave (F1) and ODTTs with each polysaccharide, as well as between ODTTs containing 1 w/w% tamarind gum with microwave (F5) and ODTTs containing 0.5, 3 and 5 w/w% tamarind gum with microwave was determined using ANOVA. *P*-values <0.05 were considered to be statistically significant.

## 3 Results and discussion

### 3.1 Evaluation of ODTTs containing polysaccharides (1 w/w% mass fraction)

**Fig. 2:**
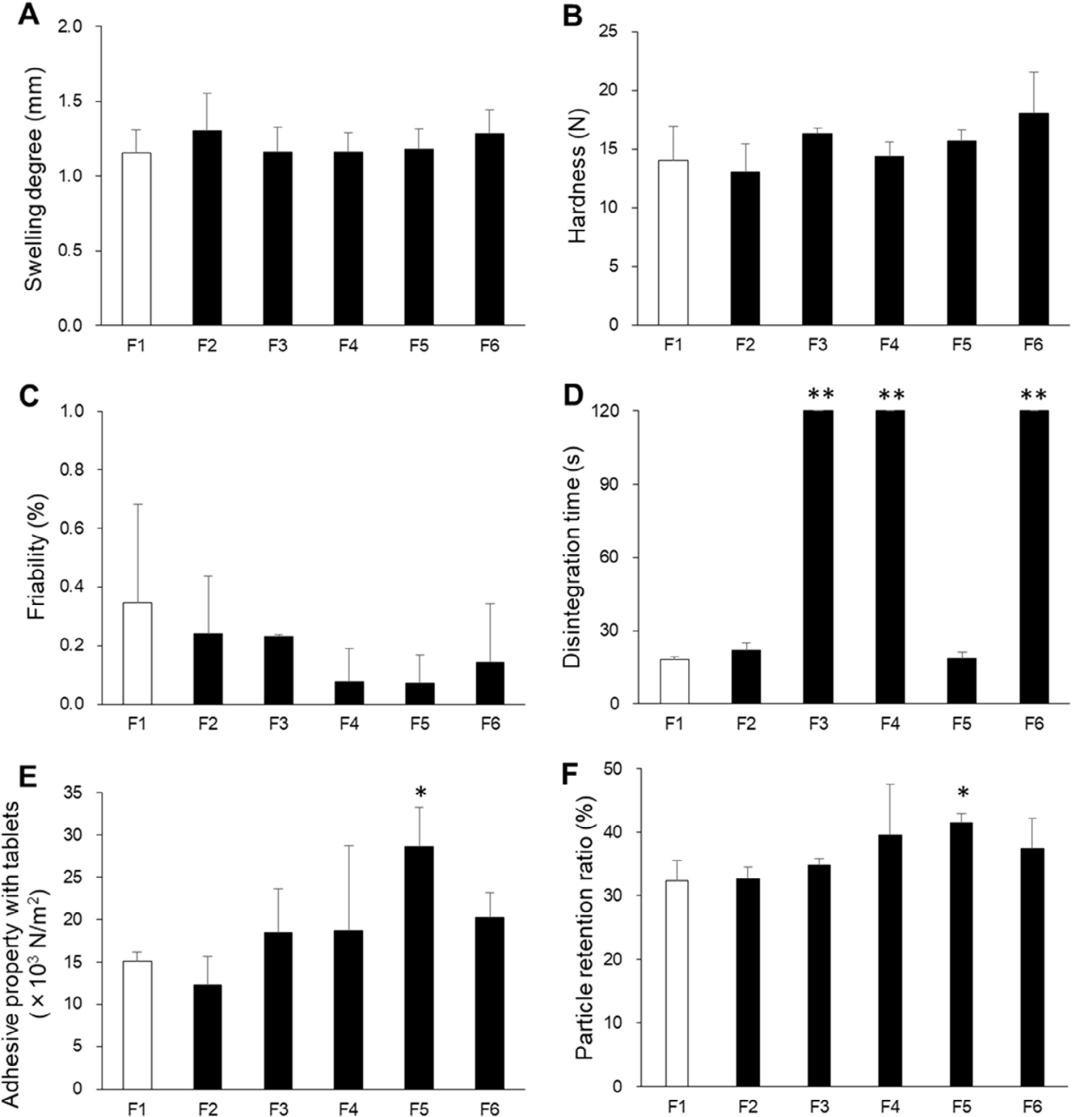
Tablet properties of orally disintegrating tea tablets manufactured using polysaccharides. (A) Swelling degree, (B) hardness, (C) friability, (D) disintegration time, (E) adhesive property with tablets, and (F) particle retention ratio. *p < 0.05 and **p < 0.01 versus the tablets without polysaccharide (F1). Each column represents the mean ± S.D. (*n* = 3).

The degree of swelling, hardness, friability, disintegration time, adhesiveness, and particle retention ratio results are shown in Fig. 2. The degree of swelling did not differ between formulations, and all formulations swelled by about 1 mm (Fig. 2A). This suggests that when the wet tablet was microwave irradiated, water vapor inside the tablet evaporated, and the tablet swelled. Sufficient swelling should shorten the disintegration time. The results of our previous study showed that swelling of over 0.2 mm was sufficient for shortening the disintegration time of ODTs (Sano et al., 2011). All formulations had hardness values of over 13 N (Fig. 2B), which is sufficient hardness for a 5-mm diameter tablet (Tanaka et al., 2016). This is because the excipient d-mannitol melts at 168 °C and forms strong particle-to-particle bonds during microwave irradiation. In addition, because of the high hardness, the friability was <1% in all formulations (Fig. 2C). However, the disintegration times were more than 120 s for F3, F4 and F6, which contained carrageenan, guar gum, and pectin, respectively (Fig. 2D). By comparison, F1 and F2, which contained gum arabic, and F5, which contained with tamarind gum, had disintegration times of <30 s. According to the Food and Drug Administration, ODTs should disintegrate within 30 s (FDA, 2008). The disintegration times of F1, F2, and F5 satisfied this requirement.

**Fig. 3:**
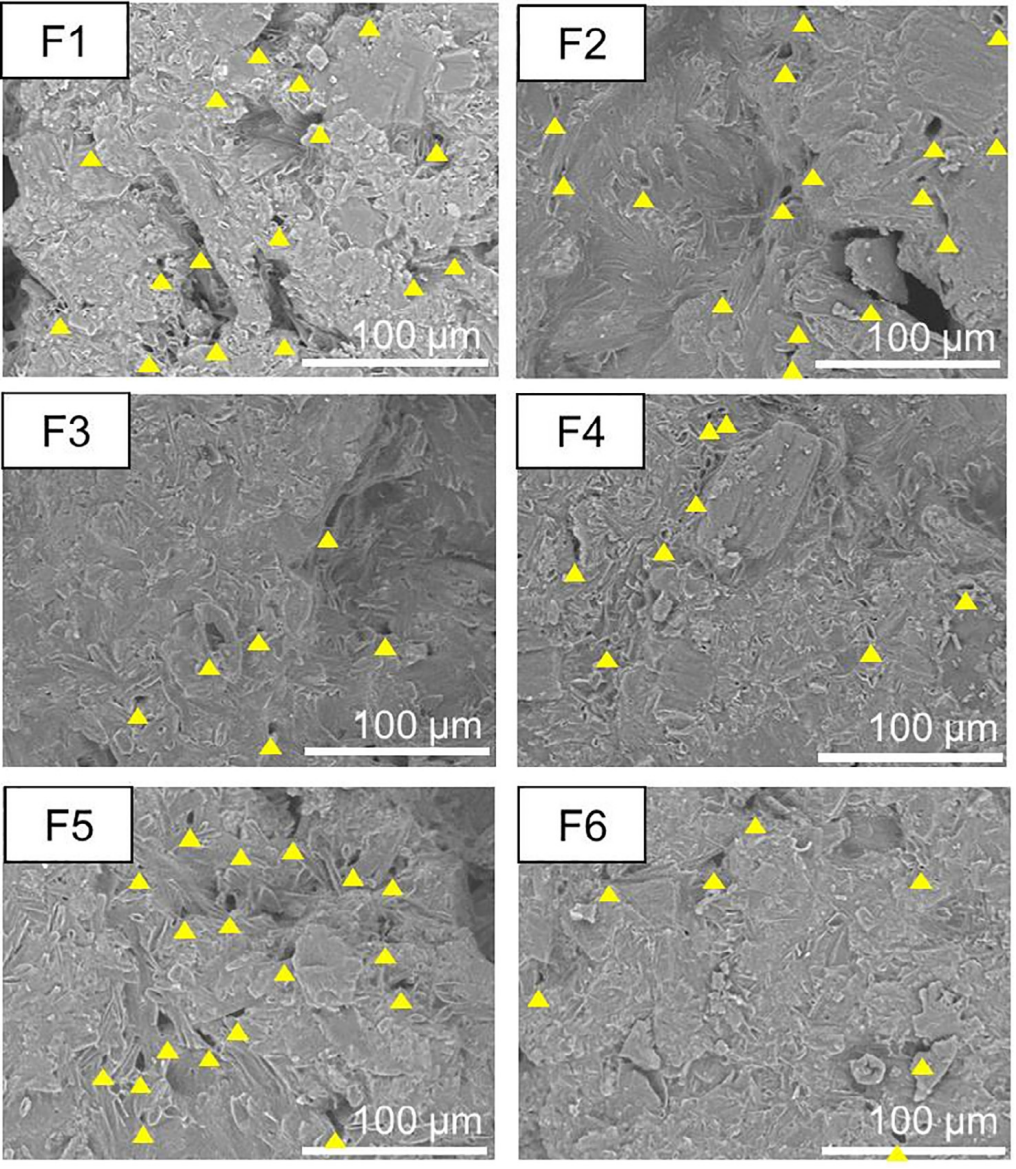
Scanning electron micrographs of the surfaces of orally disintegrating tea tablets manufactured using polysaccharides. Magnification 600× (F1, F2, F3, F4, F5, and F6). Yellow Triangles indicated the void with a diameter of over 3 μm. (For interpretation of the references to colour in this figure legend, the reader is referred to the web version of this article.)

We also observed the surfaces of the tablets by SEM (Fig. 3). Many small voids (diameter: over 3 μm) were observed in F1, F2, and F5, whereas few voids were observed in F3, F4, and F6. Generally, as the number of voids increases, the water permeation to the interior of the tablet increases and the disintegration time decreases (Kondo et al., 2012). By contrast, as the number of voids decreases, the water permeation decreases and the disintegration time increases. Therefore, the disintegration times indicate that F1, F2, and F5 have many voids and good water permeation.

**Fig. 4:**
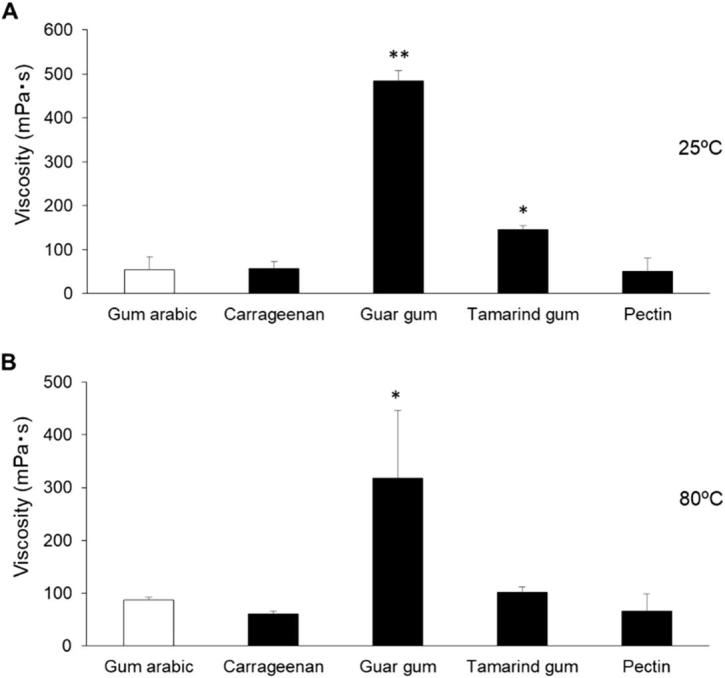
Viscosities of polysaccharides dissolved in a 1.68 M Na_2_HPO_4_ solution. (A) 25 °C and (B) 80 °C. *p < 0.05 and **p < 0.01 versus gum arabic. Each column represents the mean ± S.D. (*n* = 3).

Among the tablets, F5 showed the highest adhesiveness with the mucin layer (Fig. 2E and F). The high adhesiveness of tamarind gum might be caused by its viscosity. We measured the viscosities of the polysaccharides at 25 °C and 80 °C (Fig. 4). Guar gum and tamarind gum had higher viscosities than other polysaccharides at 25 °C. We assumed that this was caused by aggregation of polysaccharide molecules by dehydration of saccharides and alcohols, and by formation of higher-order networks by hydrogen bonding of saccharides and alcohols with polysaccharide molecules. From these results, we considered that artificial saliva rapidly infiltrated into F5 in the 20 s and the tamarind gum dissolved. The adhesiveness was high in this case because tamarind gum strongly bound to mucin via hydrogen bonding. Guar gum also had a high viscosity like tamarind gum, but the adhesiveness of the tablet containing guar gum was not as high as that of F5. We considered that this difference was caused by the permeability of the ODTTs. A small air gap was observed in F4 and it had a disintegration time of 120 s (Fig. 3), so the permeability to F4 appeared to be lower than that of F5. Therefore, despite the high viscosity, the amount of guar gum dissolved was small and the adhesiveness of F4 was lower than that of F5. For the particle retention ratio, although bonding between the pulverized tablet particles and the mucin layer was relatively high (Fig. 2F), F4 had a disintegration time of 120 s or more.

In summary, although there were no differences in the swelling degree, hardness, and friability among the formulations, F2 and F5 quickly disintegrated within 30 s and F5 had high adhesiveness properties and a high particle retention ratio.

### 3.2 Effect of the tamarind gum concentration on physicochemical properties of the ODTTs

**Fig. 5:**
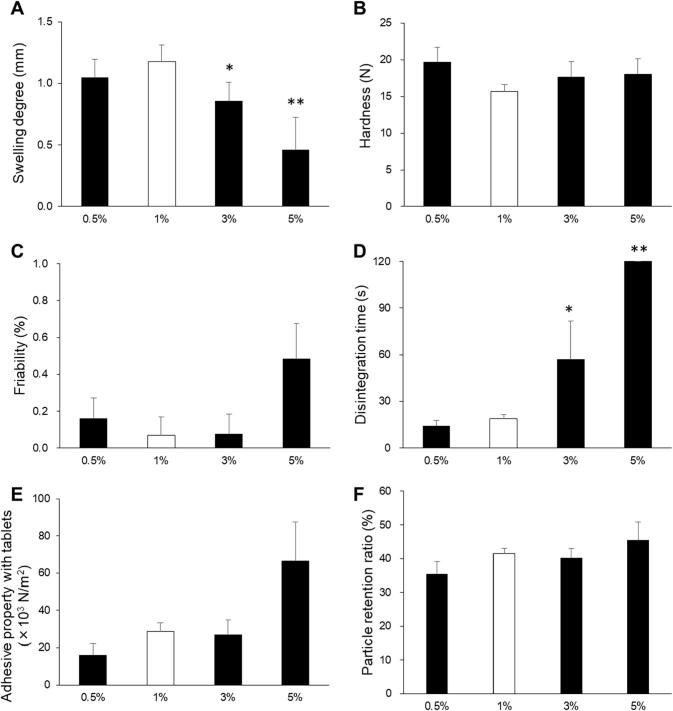
Properties of orally disintegrating tea tablets manufactured using tamarind gum. (A) Swelling degree, (B) hardness, (C) friability, (D) disintegration time, (E) adhesiveness, and (F) particle retention ratio. *p < 0.05 and **p < 0.01 versus the tablets with 1 w/w% tamarind gum. Each column represents the mean ± S.D. (*n* = 3).

Our results showed that ODTTs containing tamarind gum had good hardness, rapid disintegration, and high adhesiveness. Therefore, we investigated the effect of the tamarind gum mass fraction on physicochemical properties of the ODTTs. We measured the degree of swelling, hardness, friability, disintegration time, adhesiveness, and particle retention ratio for tablets with tamarind gum mass fractions of 0.5, 1, 3, and 5 w/w% (Fig. 5). For ODTTs with tamarind gum mass fractions of 0.5 and 1 w/w%, the degree of swelling was about 1 mm, but as the tamarind gum mass fraction increased to 3 and 5 w/w%, the degree of swelling decreased to about 0.5 mm (Fig. 5A). This is because a gel was formed in the tablet as the proportion of tamarind gum increased, and even though microwave irradiation was carried out, the water vapor could not sufficiently expand the tablet. The hardness values were over 13 N in all formulations (Fig. 5B). Because of the high hardness, all formulations had friability values of <1% (Fig. 5C), which suggests that the hardness is enough. When the tamarind gum mass fraction was 0.5 or 1 w/w%, the disintegration time was <30 s (Fig. 5D); however, as the tamarind gum mass fraction increased, the disintegration time increased to >60 s.

**Fig. 6:**
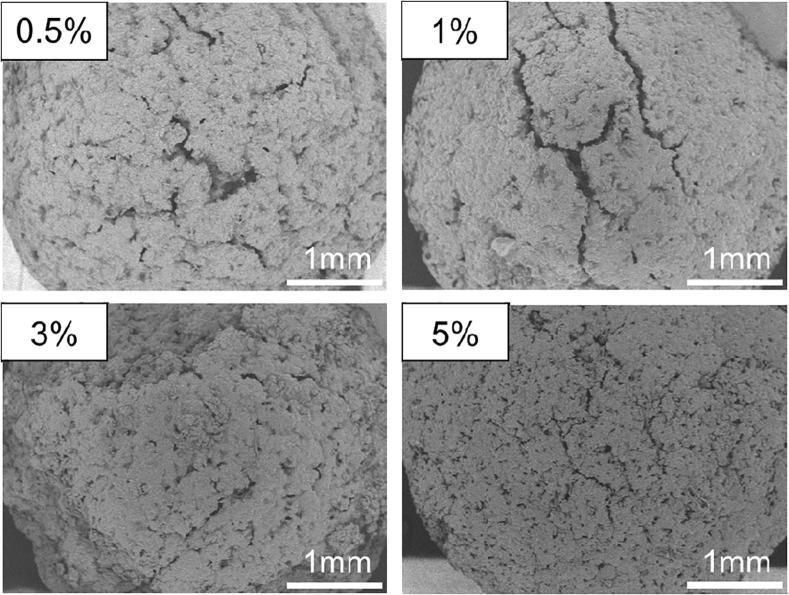
Scanning electron micrographs of the surfaces of orally disintegrating tea tablets manufactured using tamarind gum. Magnification 40× (0.5, 1, 3 and 5 w/w%).

When we observed the tablet surfaces by SEM, many voids were observed in ODTTs containing 0.5 and 1 w/w% (mass fraction) tamarind gum, whereas hardly any voids were observed in ODTTs containing 3 and 5 w/w% (mass fraction) tamarind gum (Fig. 6). As discussed above, with an increase in the viscosity of tamarind gum, the surface voids would disappear. This indicates that the lack of voids prevents water permeation into the tablet and extends the disintegration time.

The adhesive properties of the tablets increased as the mass fraction of tamarind gum increased (Fig. 5E). Because of the increase in the tamarind gum mass fraction, the viscosity increased and the bond with the mucin layer became stronger. This increased the load and the adhesiveness increased. In addition, as the mass fraction of tamarind gum increased, the particle retention ratio increased (Fig. 5F). It is thought that as the content of tamarind gum bonded to the mucin layer increased, the adhesiveness increased. Therefore, although an increase in the mass fraction of tamarind gum tended to enhance the adhesiveness of the tablet, addition of 1 w/w% tamarind gum produced tablets with sufficient hardness, disintegration, and adhesiveness.

### 3.3 Evaluation of the dissolution behavior of EGCG

**Fig. 7:**
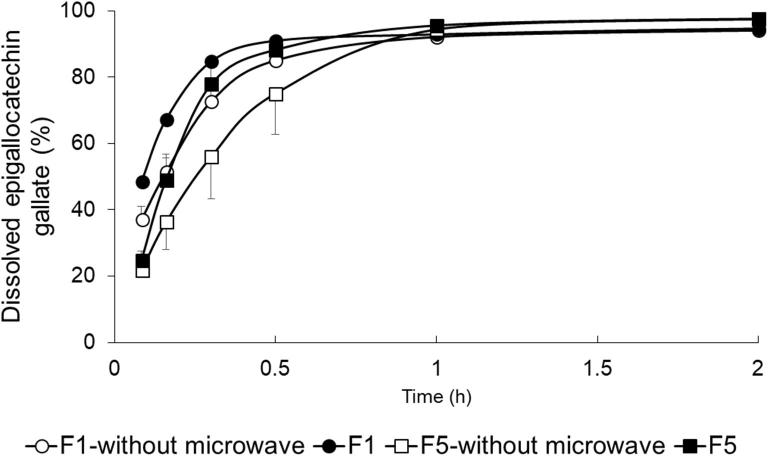
Dissolution profile of EGCG from polysaccharide-free ODTTs with/without microwave and ODTTs containing 1 w/w% (mass fraction) tamarind gum with/without microwave. Each symbol represents the mean ± S.D. (*n* = 3).

We studied the dissolution behavior of EGCG, which is an active compound in tea leaves (Fig. 7). We examined its dissolution using ODTTs containing 1 w/w% (mass fraction) tamarind gum and prepared with microwave (F5). These tablets showed rapid disintegration and high adhesiveness. In addition, we carried out a dissolution test with polysaccharide-free tablets without microwave (F1-without microwave), ODTTs with microwave (F1), and tablets containing 1 w/w% (mass fraction) tamarind gum without microwave (F5-without microwave). F1 showed the highest dissolution rate until 30 min, and after 1 h, all tablets showed dissolution rates of almost 90%. Compared with F1 and F5, even with the addition of tamarind gum, F5 showed a dissolution rate of 90% or more after 1 h. Therefore, addition of tamarind gum hardly affected the dissolution rate of EGCG. In addition, microwave -treated tablets (F1 and F5) showed rapid dissolution compared with tablets prepared without microwave. This was because the powdered tea became friable at the high temperature induced by microwave irradiation, and this increased the EGCG dissolution.

### 3.4 Quantitative analysis of catechin

In addition to the dissolution study of EGCG, we extracted other active compounds from the tablets and performed quantitative analyses for further antibacterial studies. The quantitative values for all compounds (ECG, GCG, EGC, and EGCG) (Table 3) were lower after microwave treatment than before. This indicates that they were likely converted to other compounds by microwave irradiation or decomposed. However, even when tamarind gum was added, fast dissolution was observed compared with the tablet without tamarind gum. This suggests that tamarind gum does not affect the active ingredients contents (ECG, GCG, EGC, and EGCG).

**Table 3:** Quantitative analysis of active compounds in tea powder.

	F1-witout microwave	F1	F5-without microwave	F5
ECG (µg/mL)	8.43 ± 1.21	3.31 ± 2.39^*^	6.49 ± 2.42	4.74 ± 0.21
GCG (µg/mL)	4.04 ± 0.34	1.30 ± 0.47	2.99 ± 1.44	1.09 ± 0.10
EGC (µg/mL)	82.1 ± 8.59	45.4 ± 4.19	76.4 ± 2.27	53.8 ± 10.7
EGCG (µg/mL)	620.8 ± 2.36	563.8 ± 0.66^**^	623.5 ± 2.31	579.1 ± 2.92^♯♯^

*p < 0.05 and **p < 0.01 versus F1-without microwave group. ^♯♯^*p* < 0.01 versus F5-without microwave. Each value represents the mean ± S.D. (*n* = 3).

### 3.5 Evaluation of the antibacterial effect

**Fig. 8:**
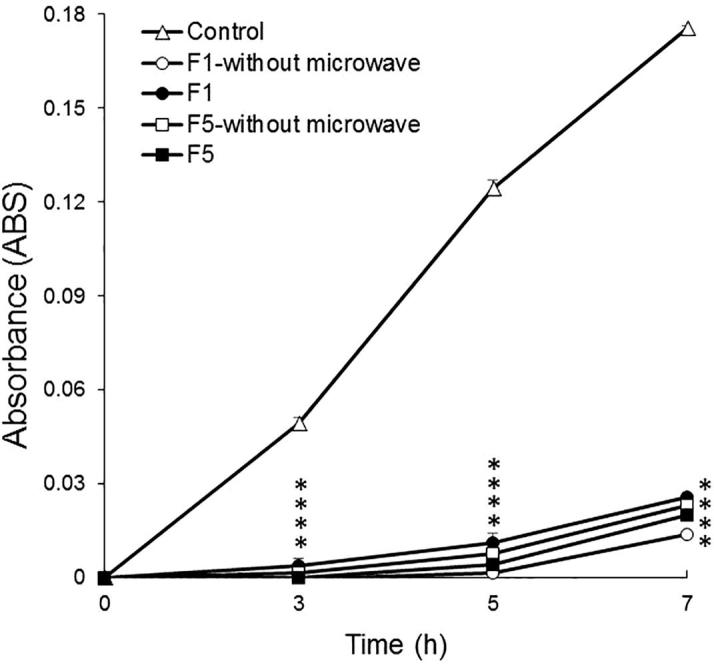
Evaluation of the antibacterial effects when extracts from tablets were treated. *p < 0.01 versus control. Each symbol represents the mean ± S.D. (*n* = 3).

Next, we studied the antimicrobial activity against *S. mutans* (Fig. 8). The vertical axis represents the absorbance at 651.5 nm. A higher absorbance value means *S. mutans* is proliferating, whereas a lower absorbance value means that growth of *S. mutans* is suppressed. The absorbance of the control gradually increased with time and marked proliferation of *S. mutans* was observed. In all formulations, the absorbances were markedly low and little bacterial growth was observed, indicating that all formulations had growth inhibitory effects. From the results of dissolution tests, we found EGCG had a dissolution rate of 90% or more at 1 h (Fig. 7). Therefore, although the amounts of the active compound in F1 and F5 were a little bit lower than those in tablets prepared without microwave irradiation, sufficient EGCG was present to show antibacterial activity. Overall, the slight differences of total amounts of EGCG did not affect the antibacterial properties. Previously, EGCG have been reported to inhibit the formation of glucosyltransferases and biofilms at 500 µg/mL (Koo et al., 2002), which is similar to our results. We can conclude that polyphenols extracted from tea leaves seems show significant growth inhibitory effects.

## 4 Conclusions

We used polysaccharides to prepare ODTTs with adhesive properties for use as novel oral care preparations. The prepared ODTTs had a swelling degree of about 1 mm, hardness of 13 N or more, and friability of 1% or less. Among the ODTTs, those containing tamarind gum disintegrated in 30 s or less because there were many voids on the tablet surface for water to penetrate. The adhesive properties and particle retention ratios were also highest in the ODTTs containing tamarind gum, which could be attributed to the rapid disintegration and high viscosity of the tamarind gum itself. When we increased the tamarind gum mass fraction in the formulation, the swelling degree decreased to about 0.5 mm and the disintegration time increased. This is because the thickening action of the gum increases as its mass fraction increases, and the tablet surface is covered by a gel. This prevents void and suppresses water penetration. Although the adhesive properties and particle retention ratios increased as the mass fraction of tamarind gum increased, the disintegration time was adversely affected. Therefore, among the mass fractions we studied, 1 w/w% tamarind gum is best for preparation of ODTTs. In our dissolution tests, even with the addition of tamarind gum, the dissolution rate was 90% or more after 1 h, indicating that addition of tamarind gum hardly affected the dissolution rate of EGCG. When we evaluated the antimicrobial activities of ODTTs with/without 1 w/w% tamarind gum, we observed growth inhibition against *S. mutans* with all formulations. In summary, ODTTs containing 1 w/w% tamarind gum have sufficient hardness, disintegration, adhesiveness, and antibacterial activity. Especially, Du et al., demonstrated that the dental adhesives containing 200 or 300 μg/mL EGCG were found to exhibit inhibitory effect on the growth of *S. mutans* (Du et al., 2012), speculating that polyphenols extracted from ODTTs with muco-adhesive property might show significant growth inhibitory effects *in vivo*. Future studies using animal models with periodontal disease are needed to determine whether the ODTTs with tamarind gum work as expected in the oral cavity. If they do, they could be used for self-medication to contribute to the health of many people.

## Declaration of interest

None.
