# Supplementary material for: Development of muco-adhesive orally disintegrating tablets containing tamarind gum-coated tea powders for oral care
Source: Int J Pharm X. 2019 Apr 4;1:100012. doi: 10.1016/j.ijpx.2019.100012 (PMC6733284; doi:10.1016/j.ijpx.2019.100012)
Supplement: Supplementary Data 1 [file mmc1.pptx]

## Slide 1
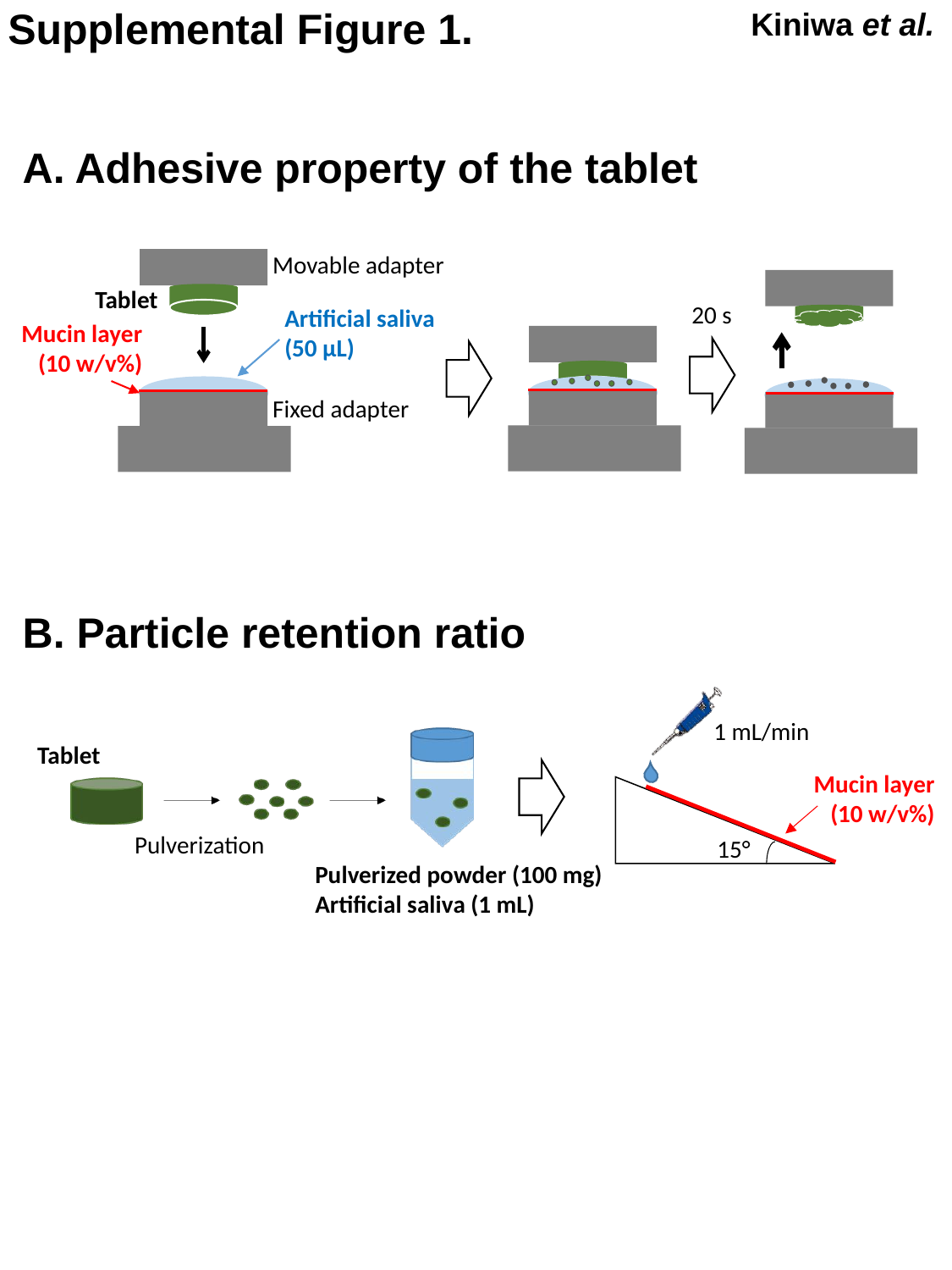

Kiniwa et al.
Supplemental Figure 1.
A. Adhesive property of the tablet
Movable adapter
Tablet
20 s
Artificial saliva
(50 µL)
Mucin layer
(10 w/v%)
Fixed adapter
B. Particle retention ratio
1 mL/min
Tablet
Mucin layer
(10 w/v%)
Pulverization
15°
Pulverized powder (100 mg)
Artificial saliva (1 mL)
